# Rho-kinase-dependent actin turnover and actomyosin disassembly are necessary for mouse spinal neural tube closure

**DOI:** 10.1242/jcs.164574

**Published:** 2015-07-15

**Authors:** Sarah Escuin, Bertrand Vernay, Dawn Savery, Christine B. Gurniak, Walter Witke, Nicholas D. E. Greene, Andrew J. Copp

**Affiliations:** 1 Newlife Birth Defects Research Centre, Institute of Child Health, University College London, WC1N 1EH, UK; 2 Cell Migration Unit, Institut für Genetik, Universität Bonn, 53115 Bonn, Germany

**Keywords:** Cytoskeleton, Embryo, Mouse, Neurulation

## Abstract

The cytoskeleton is widely considered essential for neurulation, yet the mouse spinal neural tube can close despite genetic and non-genetic disruption of the cytoskeleton. To investigate this apparent contradiction, we applied cytoskeletal inhibitors to mouse embryos in culture. Preventing actomyosin cross-linking, F-actin assembly or myosin II contractile activity did not disrupt spinal closure. In contrast, inhibiting Rho kinase (ROCK, for which there are two isoforms ROCK1 and ROCK2) or blocking F-actin disassembly prevented closure, with apical F-actin accumulation and adherens junction disturbance in the neuroepithelium. Cofilin-1-null embryos yielded a similar phenotype, supporting the hypothesis that there is a key role for actin turnover. Co-exposure to Blebbistatin rescued the neurulation defects caused by RhoA inhibition, whereas an inhibitor of myosin light chain kinase, ML-7, had no such effect. We conclude that regulation of RhoA, Rho kinase, LIM kinase and cofilin signalling is necessary for spinal neural tube closure through precise control of neuroepithelial actin turnover and actomyosin disassembly. In contrast, actomyosin assembly and myosin ATPase activity are not limiting for closure.

## Abstract (precis)

**Summary:** Actomyosin assembly and myosin ATPase activity are not essential for mouse spinal neurulation. However, the ROCK–LIMK–cofilin pathway, which controls actin turnover and actomyosin disassembly, is necessary.

## INTRODUCTION

Neurulation is the embryonic process by which the neural plate becomes converted into a closed neural tube, the developmental precursor of the brain and spinal cord. Bending of the neuroepithelium creates the neural folds whose elevation and fusion are required for neural tube formation. The role of the cytoskeleton has long been studied in neurulation. Actin microfilaments are located circumferentially in the apices of neuroepithelial cells (Sadler et al., 1982) together with non-muscle myosin II, which is recruited to this site (Kinoshita et al., 2008; Nyholm et al., 2009). Regulated actomyosin contraction is often viewed as the principal ‘motor’ that induces apical constriction, thereby causing the neural plate to bend and close. This is analogous to proposed mechanisms of other epithelial invagination events (Sawyer et al., 2010).

Apical actomyosin contraction is regulated by proteins, including RhoA and Shroom3, which localize to the apical actomyosin complex. They are linked to the function of apical junctional complexes: the sites of contractile microfilament insertion (Hildebrand, 2005; Nishimura and Takeichi, 2008). Interestingly, planar cell polarity (PCP) proteins, including Celsr1, have also been implicated in the regulation of actomyosin contractility in neural tube closure (Nishimura et al., 2012), extending the role of PCP that is known to function in early neural plate shaping (Wallingford and Harland, 2002; Ybot-Gonzalez et al., 2007b).

Although actomyosin-driven apical constriction is widely accepted to drive neurulation, several considerations suggest that this view might be overly simplistic. First, the neural plate of higher vertebrates bends focally, at the median hinge point and dorsolateral hinge points (MHP and DLHPs, respectively), rather than through generalized invagination of the whole neuroepithelium. Actin does not localize specifically to hinge points, but is present in the apices of all neuroepithelial cells (Ybot-Gonzalez and Copp, 1999). Although Shroom3 has been reported to localize to bending regions in the *Xenopus* neural plate (Haigo et al., 2003), no specific protein localization has been described for higher vertebrate hinge points.

A second challenge is that neuroepithelial-bending sites do not appear to be locations of active apical constriction. For example, the MHP of chick and mouse embryos is enriched for wedge-shaped cells (Schoenwolf and Franks, 1984; Smith et al., 1994), but this results from a localized prolongation of the cell cycle that causes nuclei to become basally located within the pseudostratifed neuroepithelium (Smith and Schoenwolf, 1988). The apices of MHP cells appear to narrow passively as a secondary result of basal cell expansion.

A third line of evidence comes from experimental disruption of the cytoskeleton during closure. In the chick, the actin-microfilament inhibitor cytochalasin D (CytD) blocks only the later stages of neurulation and considerable neural fold elevation occurs in its presence (Schoenwolf et al., 1988). In mammals, although cytochalasins are potent inhibitors of cranial neural tube closure, both *in vivo* (Austin et al., 1982; Wiley, 1980) and in cultured embryos (Morriss-Kay and Tuckett, 1985), spinal neurulation is resistant to CytD. Following actin microfilament disassembly in cultured mouse embryos, closure progresses and the MHP and DLHPs continue to form (Ybot-Gonzalez and Copp, 1999). Consistent with this, cytochalasins fail to induce spina bifida when administered *in vivo* (Austin et al., 1982; Wiley, 1980).

Finally, the findings with cytoskeletal inhibitors are mirrored in studies of mice with null mutations in cytoskeletal genes. Cranial NTDs (exencephaly) are seen in mutants lacking a single cytoskeletal protein [e.g. palladin, vinculin, cofilin 1 (CFL1) and MARCKS], in double and triple mutants of actin regulatory proteins (e.g. MENA, VASP and EVL), and in mice lacking protein kinases with cytoskeletal influence (e.g. ABL1 and ABL2, and MAPK8 and MAPK9) (Copp and Greene, 2010). In contrast, spinal neurulation occurs normally in such embryos. Only knockouts of the cytoskeleton-associated proteins Shroom3 and MARCKS-related protein (Hildebrand and Soriano, 1999; Wu et al., 1996) yield both exencephaly and spina bifida and, even here, the frequency of exencephaly exceeds spina bifida. Hence, although the actomyosin cytoskeleton appears essential for cranial neurulation, its role in spinal closure is unclear.

Here, we examined in detail the role of the cytoskeleton in mouse spinal neural tube closure. Inhibitors were used to block specific cytoskeletal events in cultured embryos whereas closure was also examined in mice genetically null for the cytoskeletal protein cofilin 1. We show that actomyosin assembly and ATPase-dependent myosin contractile function are not limiting in spinal neural tube closure. In contrast, there is an essential role for F-actin turnover with the disassembly of actomyosin complexes being a crucial step that, when inhibited, leads to severe compromise of neurulation.

## RESULTS

### Inhibition of RhoA signalling disrupts the progression of spinal neural tube closure

**Fig. 1.**
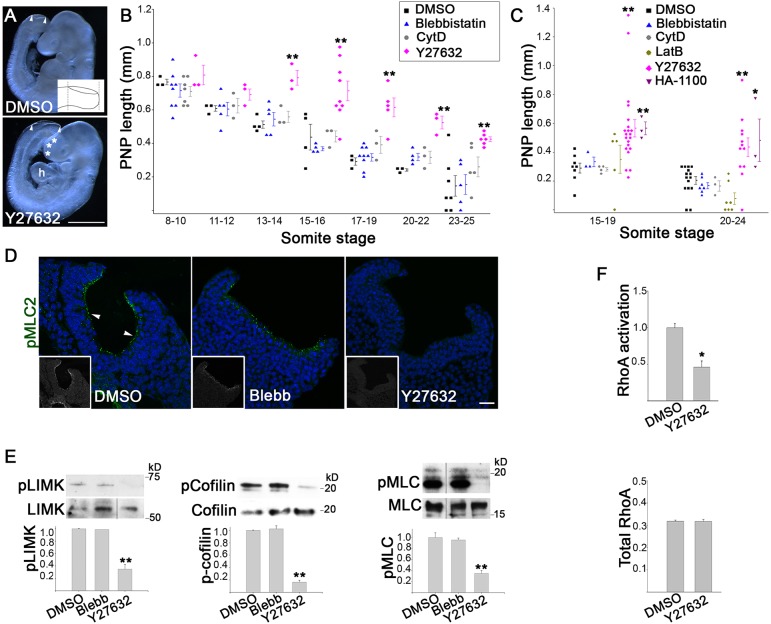
**Inhibition of RhoA signalling disrupts spinal neural tube closure.** (A) Mouse embryos cultured for 18 h to the 24 somite stage. Exposure to 5 µM Y27632 yields an enlarged PNP (between arrowheads), open forebrain (asterisks) and defective heart looping (h) compared with the DMSO control. Inset, method of PNP length measurement, between the dashed lines. Scale bar: 0.5 mm. (B) Progressive closure of PNP is arrested after the 13 somite stage in embryos cultured for 5–6 h in Y27632 (**P<0.001 versus DMSO). Embryos exposed to 50 µM Blebbistatin or 0.05 µg/ml CytD close at the same rate as DMSO controls. (C) PNP closure is significantly delayed after culture in ROCK inhibitor Y27632 or HA-1100, for 18–20 h to the 15–19 or 20–24 somite stage (**P<0.001 versus DMSO). Embryos cultured in Blebbistatin, CytD or LatB resemble DMSO controls. (D) Immunohistochemistry (green, anti-pMLC; nuclei, DAPI) shows apically localized pMLC (arrowheads) in DMSO- and Blebbistatin-treated embryos with 19 somites. The signal is abolished by 5–6 h culture in Y27632. Grayscale insets, pMLC staining only. Scale bar: 30 µm. (E) Western blots for pLIMK and LIMK, p-cofilin and cofilin, and pMLC and MLC. The level of the phosphorylated forms are quantified as a proportion of the total, after normalizing to GAPDH. Culture for 5–6 h in Y27632 significantly reduces the relative amount of pMLC, pLIMK and p-cofilin compared with DMSO controls, whereas Blebbistatin has no effect (*n*=3, **P<0.001). (F) G-LISA assay of active GTP-bound RhoA (top) relative to total RhoA (below), normalized to DMSO (mean±s.d.). Y27632-treated embryos have significantly reduced RhoA activation whereas total RhoA does not differ between treatments (*n*=3, *P<0.001). Significance values were calculated with a Student's *t*-test, compared with DMSO control.

Mouse embryos were explanted between embryonic days (E) 8.5 and 9.5, after neural tube closure had been initiated. Following culture for 5–6 h in the presence of cytoskeletal inhibitors, the length of spinal axis occupied by open neural folds (the posterior neuropore; PNP; Fig. 1A) was taken as a measure of neural tube closure progression. PNP enlargement indicates neurulation delay or cessation and predisposition to spina bifida (Copp, 1985).

Dose–response studies were used to select inhibitor concentration: we generally used the highest concentration at which general embryonic toxicity was not observed (supplementary material Table S1). CytD, a drug preventing polymerization of actin monomers (Flanagan and Lin, 1980), induced exencephaly at high frequency (not shown) but had no effect on spinal closure (Fig. 1B) consistent with previous findings (Wiley, 1980; Ybot-Gonzalez and Copp, 1999). Moreover, treatment with Blebbistatin which blocks myosin II in an actin-detached state and prevents actomyosin cross-linking (Kovacs et al., 2004), also induced exencephaly but did not affect spinal closure (Fig. 1B), even when assessed over a fourfold concentration range (supplementary material Fig. S1A,D). In marked contrast, treatment with Y27632, a selective inhibitor of Rho kinase (ROCK, for which there are two isoforms ROCK1 and ROCK2) (Uehata et al., 1997), delayed spinal closure. After only 5–6 h exposure, Y27632-treated embryos had significantly enlarged PNPs compared with DMSO controls (Fig. 1A,B; supplementary material Fig. S1B), consistent with a report of neurulation disturbance by Y27632 in chick embryos (Wei et al., 2001).

To test whether Blebbistatin might require more prolonged exposure to be effective, or whether Y27632 had only a temporary effect, we cultured embryos for 18–20 h, to the 15–19 or 20–24 somite stage. All three inhibitors gave a similar result as in the short-term exposure: CytD and Blebbistatin did not affect spinal closure whereas Y27632 produced enlarged PNPs (Fig. 1C; supplementary material Fig. S1B). Even longer culture, to the 25–36 somite stage when PNP closure is usually completed, yielded closed PNPs in embryos exposed to CytD (9/10 closed), Blebbistatin (7/7) and DMSO (12/12), but persistently open PNPs in Y27632-treated embryos (0/14 closed).

To replicate the effects of CytD and Y27632, we cultured embryos in additional inhibitors: Latrunculin B (LatB), an inhibitor of actin polymerization (Spector et al., 1989), had no effect on PNP closure, like CytD (Fig. 1C; supplementary material Fig. S1B), whereas hydroxyfasudil (HA-1100), another ROCK inhibitor (Shimokawa et al., 1999), delayed PNP closure in a manner that was very similar to Y27632 (Fig. 1C; supplementary material Fig. S1B). We conclude that ROCK inhibition (either by Y27632 or HA-1100) blocks the progression of mouse spinal neurulation, whereas CytD, LatB and Blebbistatin have no effect.

### Abnormal actomyosin accumulation in ROCK-inhibitor-treated embryos

Effectiveness and specificity of inhibitors was assessed by immunoblotting and immunohistochemistry. Y27632 inhibits ROCK-dependent phosphorylation of myosin light chain (MLC), LIM domain kinase (LIMK, for which there are two isoforms LIMK1 and LIMK2) and cofilin (here, we use an antibody that recognises cofilin 1 and cofilin 2; however, as we study an embryonic region lacking cofilin 2, we detect only cofilin 1) (Maekawa et al., 1999), and we detected marked diminution of phosphorylated MLC (pMLC), phosphorylated LIMK (pLIMK) and phosphorylated cofilin (p-cofilin) in Y27632-treated embryos, but not in those exposed to Blebbistatin (Fig. 1D,E). Using a G-LISA assay, we detected a reduction of RhoA activity in Y27632-treated embryos (Fig. 1F), consistent with previous findings (Tang et al., 2012).

**Fig. 2.**
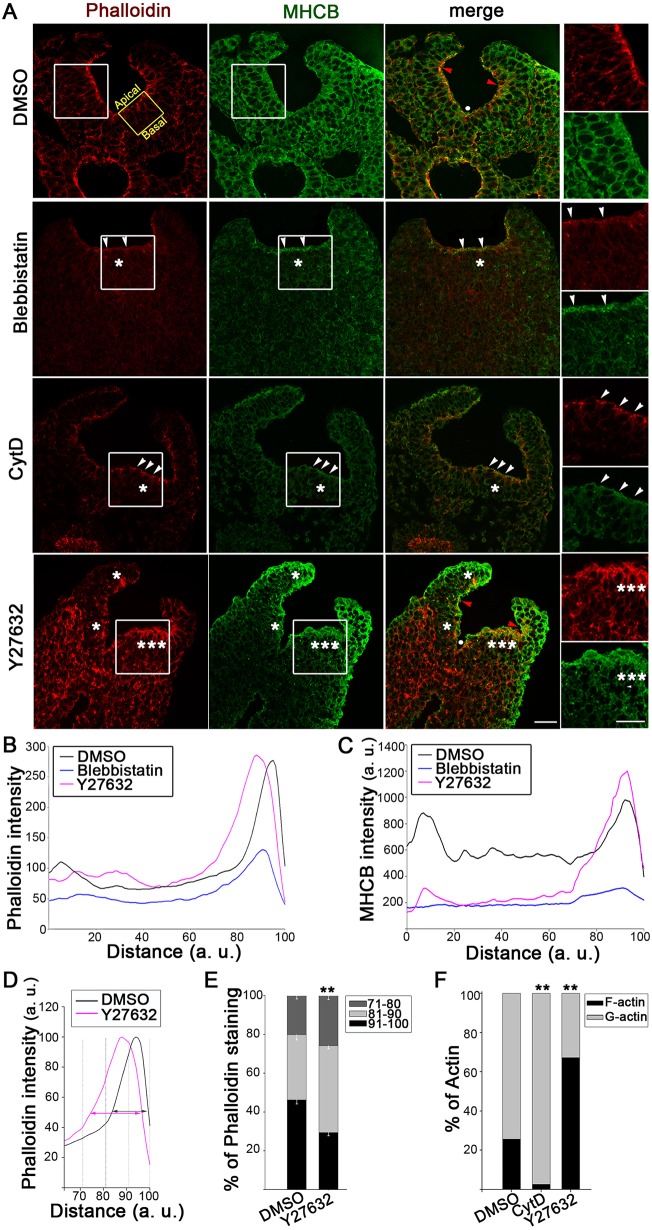
**ROCK inhibition leads to actomyosin accumulation in apical neuroepithelium.** (A) Immunohistochemistry for F-actin (phalloidin, red) and myosin II (anti-MHCB, green). Culture for 5–6 h in CytD or Blebbistatin strongly reduces apical (arrowheads) and non-apical (single asterisks) staining. In contrast, actomyosin appears abnormally extensive after Y27632 treatment (multiple asterisks). Note the presence of the MHP (white dot) and DLHPs (red arrowheads) in both Y27632-treated and DMSO control embryos. Right, enlargement of the boxed areas. The yellow box in top left image shows the typical area of neuroepithelium in which basal-to-apical staining intensity was measured. Embryos shown have 17–19 somites. Scale bars: 30 µm. (B,C) Intensity profile scans of phalloidin (B) and anti-MHCB (C) staining along the neuroepithelial basal-to-apical axis. Basal surface, 0; apical surface, 100 (a.u., arbitrary units). F-actin is enhanced apico-basally and MHCB is redistributed to the apical surface in Y27632-treated embryos compared with DMSO controls. Blebbistatin reduces both F-actin and MHCB. (D,E) With maximum intensity normalized to 100%, the apical domain of phalloidin staining extends farther basally in Y27632-treated embryos than in DMSO controls (arrows in D). Quantification in the three apical neuroepithelial bins (71–80%, 81–90%, 91–100%) shows that phalloidin extends significantly more basally in Y27632-treated embryos compared with DMSO (**P<0.001 in E). (F) Biochemical fractionation of F- and G-actin. Stable F-actin represents 70% of actin in Y27632-treated embryos, only 25% in DMSO controls and less than 3% after CytD treatment (**P<0.001). Significance values were calculated with a Student's *t*-test, compared with DMSO control.

Phalloidin staining of neuroepithelial sections (Fig. 2A) revealed an organized actin network in DMSO-treated control embryos, with intense signal at the apical surface and aligned microfilaments within the neuroepithelium. Immunohistochemistry for non-muscle myosin II heavy chain B (MHCB, also known as MYH10; Fig. 2A) showed more generalized cellular staining, with marked colocalization of actin and myosin II at the apical neuroepithelial surface (Fig. 2A, merge). Both actin and myosin II distribution were severely disrupted in embryos treated with CytD or Blebbistatin: only a narrow domain of actomyosin remained visible in the most apical part of the neuroepithelium (Fig. 2A). We quantified actin and myosin II staining using a confocal-based scanning method to provide a basal-to-apical profile of staining intensity for each neuroepithelium (see supplementary material Fig. S1E). This showed a striking diminution of both actin and myosin II staining in the Blebbistatin-treated neuroepithelium, particularly in the apical region, compared with DMSO (Fig. 2B,C). Combined exposure to Blebbistatin and CytD further diminished actin and myosin immunostaining (supplementary material Fig. S2C,D), but these embryos also showed normal PNP closure (supplementary material Fig. S1D). Hence, the lack of effect of CytD and Blebbistatin on spinal neural tube closure occurs despite a major disruption of actomyosin in the closing neuroepithelium.

Y27632 treatment led to a dramatic enhancement of phalloidin and anti-MHCB staining at the apical surface of the neuroepithelium (Fig. 2A). Quantification showed an expansion of the average apical intensity peak of phalloidin towards the basal neuroepithelial surface (Fig. 2B), which was highly reproducible between embryos (supplementary material Fig. S2A). Dividing the apical portion of the neuroepithelium into three bins, at 71–80%, 81–90% and 91–100% of the normalized basal-to-apical distance (Fig. 2D), we found a significantly greater proportion of total actin in the more basal bins after Y27632 treatment (Fig. 2D,E). Biochemical fractionation showed that ∼70% of total actin is filamentous in Y27632-treated embryos, whereas only 25% is F-actin in DMSO controls (Fig. 2F; supplementary material Fig. S2B). In contrast, CytD-treated embryos contained extremely little F-actin, with the great majority being soluble G-actin (Fig. 2F; supplementary material Fig. S2B). Anti-MHCB staining intensity was also markedly redistributed to the apical neuroepithelial surface after Y27632 (Fig. 2A,C). We conclude that ROCK inhibition causes an abnormal actomyosin accumulation apically within the neuroepithelium.

### Increased F-actin stabilization is sufficient to delay spinal neural tube closure

**Fig. 3.**
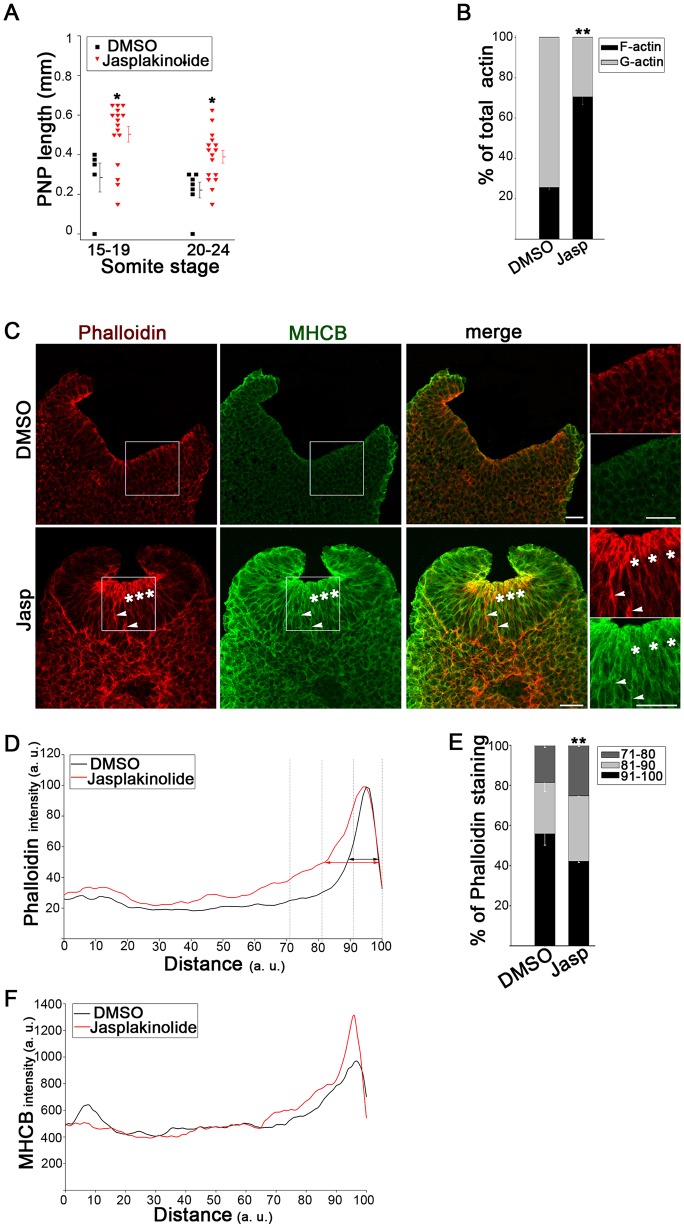
**Stabilization of F-actin delays spinal neural tube closure.** (A) PNP length is significantly increased at the 15–19 and 20–24 somite stage after 18–20 h culture in 10 nM jasplakinolide (Jasp), compared with DMSO controls (*P<0.05). (B) Biochemical fractionation shows proportionately increased F-actin and reduced G-actin in Jasp-treated embryos relative to DMSO controls (**P<0.001). (C) Immunohistochemistry (phalloidin, red; anti-MHCB, green) reveals actomyosin accumulation at the apical neuroepithelial surface (asterisks) and on some lateral cell surfaces (arrowheads) after Jasp treatment (embryos have 20–21 somites). Right, enlargement of the boxed areas. Scale bars: 30 µm. (D–F) Intensity profile scans along the neuroepithelial basal-to-apical axis. Jasp-treated embryos show an extension of phalloidin staining (intensity normalized to 100%) towards the basal surface (arrows in D), which is confirmed by quantification in the most apical 30% of the neuroepithelium (E; **P<0.001). MHCB staining intensity (non-normalized) is greater apically in Jasp-treated embryos than in DMSO controls (F). Significance values were calculated with a Student's *t*-test, compared with DMSO control.

To test whether apical actomyosin accumulation, as seen after Y27632 treatment, might be responsible for defective spinal neurulation we used Jasplakinolide (Jasp), a drug that blocks actin depolymerization and increases the F-actin pool (Cramer, 1999). Culture in Jasp for 18–21 h to the 15–19 and 20–24 somite stages caused significant closure delay compared with DMSO controls (Fig. 3A; supplementary material Fig. S2E). More than 70% of actin was filamentous in these embryos (Fig. 3B) with a threefold reduction in monomeric G-actin (supplementary material Fig. S2F). Sections showed a massive accumulation of colocalized F-actin and MHCB at the apical surface as well as patches more basally within the neuroepithelium (Fig. 3C). Quantification confirmed the mainly apical accumulation of actomyosin (Fig. 3D–F), which was reproducible between embryos (supplementary material Fig. S2G). We considered the possibility that inhibition of ROCK might lead to a RhoA-dependent inhibition of embryonic cell proliferation or increase in cell death. However, measurement of the percentage of neuroepithelial cells positive for phosphorylated histone H3 staining and activated caspase 3 showed that neither cell proliferation nor programmed cell death was altered in Y27632- or Jasp-treated embryos (supplementary material Fig. S2H,I). We conclude, therefore, that F-actin stabilization by Jasp is sufficient to cause apical actomyosin accumulation and to inhibit spinal neural tube closure.

### RhoA signalling requirement for neurulation is independent of myosin II contractility

**Fig. 4.**
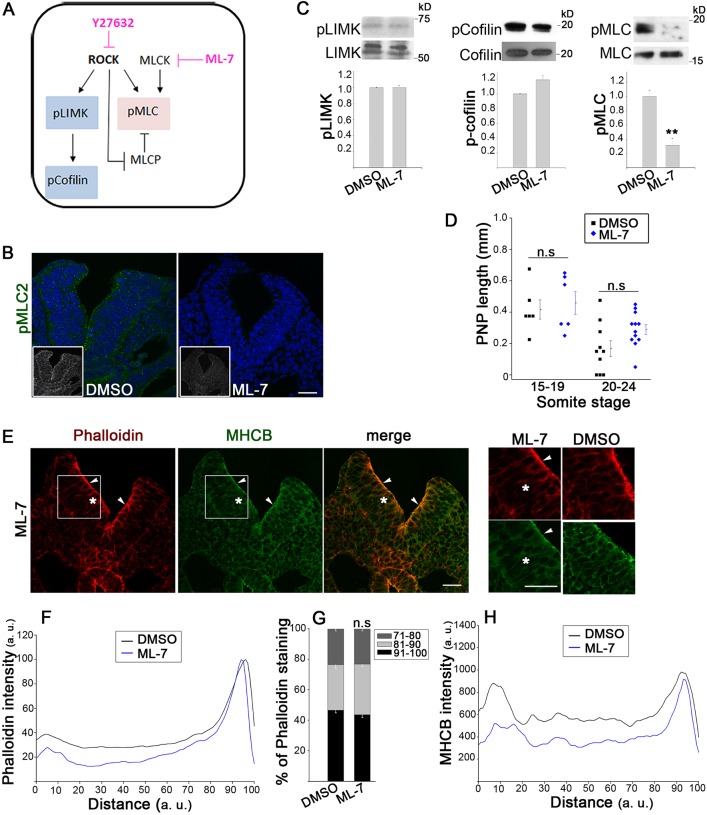
**Spinal neurulation proceeds despite inhibition of myosin II contractility.** (A) Downstream effectors of ROCK and inhibitory action of Y27632 and ML-7. (B) Immunohistochemistry shows absence of pMLC (green; nuclei, DAPI) from apical neuroepithelium of ML-7-treated embryos, compared with DMSO controls (embryos have 18–19 somites). Grayscale insets, pMLC staining only. Scale bar: 30 µm. (C) Western blots for pLIMK and LIMK, p-cofilin and cofilin, and pMLC and MLC. Embryos cultured for 5–6 h in ML-7 have significantly reduced the amount of pMLC compared with that in DMSO controls, whereas pLIMK and p-cofilin are unaffected (*n*=3, **P<0.001). (D) Culture for 5–6 h in 50 µM ML-7 does not significantly affect closure compared with DMSO at the 15–19 somite stage (n.s., *P*>0.05). (E) Immunohistochemistry (phalloidin, red; anti-MHCB, green) of ML-7-treated embryos shows apical actomyosin closely resembling DMSO controls (arrowheads; see insets on right), whereas actomyosin is reduced more basally (asterisks). Embryos have 18 somites. Scale bars: 30 µm. (F–H) Intensity profile scans show unchanged apical phalloidin staining after ML-7 treatment (F), as confirmed by quantification (G; n.s, *P*>0.05 versus DMSO). The more-basal phalloidin (F) and MHCB (H; non-normalized) staining is reduced by ML-7. Significance values were calculated with a Student's *t*-test, compared with DMSO control.

Downstream of RhoA, ROCK regulates phosphorylation of LIMK and MLC (Fig. 4A), with pMLC being a key regulatory component of myosin II ATPase activity in cellular constriction (Matsumura, 2005). To investigate this pathway, we used ML-7, a specific inhibitor of MLC kinase (Saitoh et al., 1987). Apical pMLC immunostaining was abolished by culture for 5–6 h in 50 µM ML-7 (Fig. 4B) and only the levels of pMLC were reduced in immunoblots of ML-7-treated embryo extracts, whereas the amounts of pLIMK and p-cofilin were unaffected (Fig. 4C). Despite these effects, ML-7 treatment was compatible with early spinal neural tube closure in embryos over a fivefold concentration range (Fig. 4D; supplementary material Fig. S3A,B). Embryos treated with ML-7 had a reduced overall intensity of F-actin and MHCB immunostaining, whereas the apico-basal distribution was very similar to that in DMSO controls (Fig. 4E–H). Embryos co-treated with ML-7 and Blebbistatin closed their PNPs normally (supplementary material Fig. S1D), despite a dramatic reduction in phalloidin and MHCB staining (supplementary material Fig. S2C,D). Hence, the requirement for RhoA signalling in spinal neurulation is unlikely to be mediated through contractility that is dependent on myosin II ATPase.

### Preventing actomyosin accumulation by Blebbistatin rescues Y27632-induced defects

**Fig. 5.**
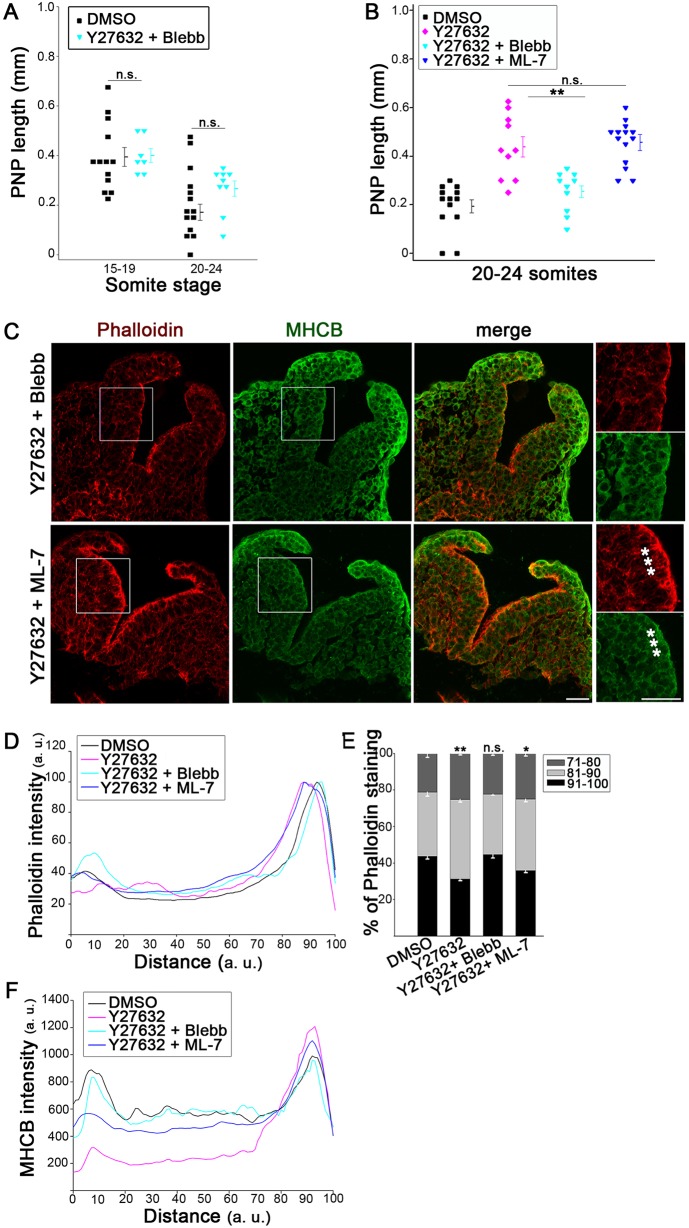
**Prevention of Y27632-related closure defects by Blebbistatin but not ML-7.** (A,B) The delayed PNP closure seen after culture in 5 µM Y27632 (B) is rescued by co-exposure to 50 µM Blebbistatin (A,B; **P<0.001) but not by ML-7 (B; n.s. *P*>0.05). Cultures were for 5–6 h (A) or 15–18 h (B), ending at the somite stages indicated. (C) Immunohistochemistry (phalloidin, red; anti-MHCB, green) reveals a normal actomyosin distribution in embryos co-exposed to Y27632+Blebbistatin (upper panels; compare with DMSO in Fig. 2A and Fig. 3C). In contrast, embryos co-exposed to Y27632+ML-7 (lower panels) show actomyosin accumulation apically (asterisks), as in those treated with Y27632 alone (see Fig. 2A). Embryos have 21 somites. (D–F) Intensity profile scans confirm the expanded apical phalloidin staining in embryos treated with Y27632 alone, and Y27632+ML-7, whereas those exposed to Y27632+Blebbistatin resemble DMSO controls (D). This is confirmed by quantification (E; **P<0.001; *P<0.05; n.s., *P*>0.05 compared with DMSO). The MHCB staining profile is very similar in Y27632+Blebbistatin and DMSO controls, but markedly abnormal in those treated with Y27632 alone and Y27632+ML-7 (F). Significance values were calculated with a Student's *t*-test, compared with DMSO control or indicated sample.

We asked whether preventing the abnormal accumulation of actomyosin in Y27632-treated embryos might ameliorate their spinal neurulation defects. Simultaneous exposure to Y27632 and Blebbistatin, or 5–6 h in Y27632 followed by 13–15 h in Blebbistatin, produced embryos with PNP lengths that were very similar to that in DMSO-treated controls (Fig. 5A,B; supplementary material Fig. S3C–E). Moreover, co-treatment with Y27632 plus Blebbistatin yielded a largely normal actomyosin distribution (Fig. 5C), which was confirmed by quantification (Fig. 5D–F). The Jasp inhibitory effect on PNP closure also appeared to be abrogated by co-exposure to Blebbistatin (supplementary material Fig. S3J,K). In contrast, embryos co-treated with Y27632 plus ML-7 showed delayed PNP closure like those exposed to Y27632 alone (Fig. 5B; supplementary material Fig. S3C) with abnormal actomyosin accumulation (Fig. 5C–F). These results argue that faulty neural tube closure in Y27632-exposed embryos resulted from abnormal actomyosin accumulation, and not elevated contractile myosin activity.

We explored in more detail the mechanism by which Blebbistatin can rescue embryos from the effects of ROCK inhibition. Short-term culture (5–6 h) in Y27632 followed by culture without inhibitor (‘washout’) yielded normal PNP lengths (supplementary material Fig. S3F,H), suggesting that the apparent rescue by Blebbistatin might be due simply to Y27632 removal. However, long-term culture (13–15 h) in Y27632 had a persistent adverse effect on PNP closure, even when the inhibitor was removed, whereas culture in Blebbistatin following long-term exposure to Y27632 yielded a normal PNP length (supplementary material Fig. S3F,H). Moreover, both short-term culture and long-term culture in Y27632 followed by continued culture in Y27632 plus Blebbistatin rescued PNP closure at different somite stages (supplementary material Fig. S3G,H). Embryos from this latter experiment had a normal F-actin:G-actin ratio (supplementary material Fig. S3I). Hence, Blebbistatin can rescue PNP closure both in the absence and presence of Y27632, arguing that it might prevent ROCK-inhibitor-dependent actomyosin accumulation and might favour actomyosin disassembly after it has accumulated.

### A key role for cofilin in regulating actomyosin accumulation and spinal neurulation

**Fig. 6.**
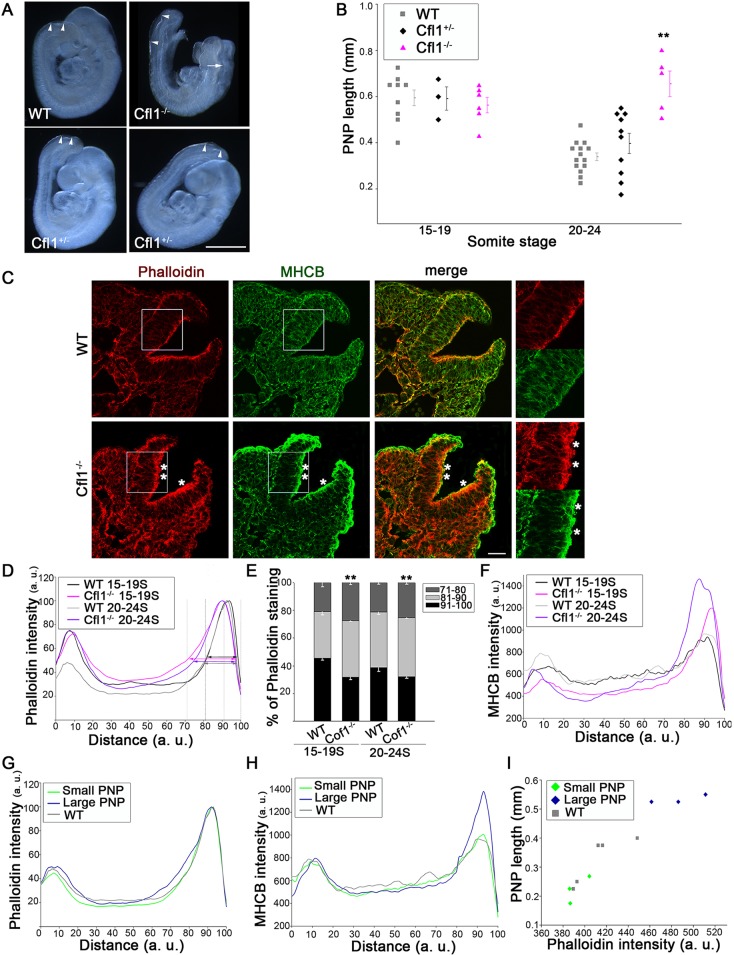
**Requirement for RhoA signalling is through the LIMK and cofilin pathway.** (A) E9.5 embryos from *Cfl1^+/−^*×*Cfl1^+/−^* mating. Homozygotes (top right) are smaller than wild-type (WT), and exhibit exencephaly (arrow) and enlarged PNP (arrowheads). Heterozygotes (lower panels) appear normal, except some have an enlarged PNP. Scale bar: 0.5 mm. (B) PNP length is significantly increased at 20–24 somites, but not at 15–19 somites, in *Cfl1^−/−^* mutants compared with WT and heterozygotes (**P<0.001). (C) Immunohistochemistry (phalloidin, red; anti-MHCB, green) shows apical actomyosin accumulation in *Cfl1^−/−^* mutants (asterisks) but not in WT, at 21–22 somites. Right: enlargement of the boxed regions. Scale bar: 30 µm. (D–F) Intensity profile scans show that phalloidin staining extends more basally in *Cfl1^−/−^* embryos than in WT (arrows in D; intensity normalized to 100%), which is confirmed by quantification (E; **P<0.001). MHCB is redistributed to the apical surface in *Cfl1^−/−^* embryos at both 15–19 and 20–24 somites (F; not normalized). (G,H) Phalloidin staining extends more basally (G) and MHCB staining is more intense apically (H) in *Cfl1* heterozygous embryos with enlarged PNPs, whereas those with small PNPs resemble WT. (I) Phalloidin staining intensity in the 71–80% basal-to-apical bin of neuroepithelium (see D) correlates with PNP length in individual *Cfl1^+/−^* (green and blue symbols) and WT embryos. Significance values were calculated with a Student's *t*-test, compared with WT control.

Cofilin-mediated actin severing activity (Bamburg, 1999) is regulated by RhoA and ROCK, through LIMK-mediated phosphorylation of cofilin (Fig. 4A). We asked whether cofilin 1 (*Cfl1*)*-*knockout embryos display reduced neuroepithelial F-actin turnover, similar to Y27632- and Jasp-treated embryos. *Cfl1^−/−^* embryos have cranial neurulation defects (Gurniak et al., 2005), as confirmed here (Fig. 6A). During spinal neurulation, PNP length did not differ between *Cfl1^−/−^* and wild-type at 15–19 somites whereas, by 20–24 somites, *Cfl1^−/−^* embryos had significantly longer PNPs than wild-type or heterozygotes (Fig. 6A,B; supplementary material Fig. S4A).

*Cfl1^−/−^* mutants with 20–24 somites exhibited abnormal actin accumulation and a marked increase in MHCB immunostaining at the apical neuroepithelium (Fig. 6C). Importantly, actomyosin accumulation was already present at the 15–19 somite stage (supplementary material Fig. S4B), before a difference in PNP length became evident. Quantification of actin staining showed a basal expansion of the apical neuroepithelial intensity peak, at both 15–19 and 20–24 somites (Fig. 6D,E). Myosin II staining intensity was moderately increased in the apical neuroepithelium at 15–19 somites, and massively increased at 20–24 somites (Fig. 6F). Hence, *Cfl1* mutants accumulate actomyosin at the neuroepithelial surface, similar to Y27632- and Jasp-treated embryos, before the onset of defective neural tube closure. Moreover, treatment of cultured *Cfl1^−/−^* embryos with Blebbistatin was able to partially restore normal actomyosin distribution (supplementary material Fig. S4G) and to markedly reduce PNP length (mean=0.43 mm; *n*=2) compared with untreated mutants (mean=0.66 mm; *n*=5). This confirms the importance of regulating actomyosin accumulation for spinal neurulation.

Mean PNP length did not differ between *Cfl1* heterozygotes and wild-type at 20–24 somites (Fig. 6B; supplementary material Fig. S4A). However, four out of ten heterozygotes had PNP lengths in excess of 0.5 mm, overlapping with *Cfl1^−/−^* mutants (Fig. 6B). Strikingly, actomyosin was more intense and expanded at the apical neuroepithelium in heterozygotes with large PNPs, whereas actomyosin staining was similar to wild-type when the PNP was small (Fig. 6G,H; supplementary material Fig. S4C,D). In fact, actomyosin staining intensity in the sub-apical region of neuroepithelium was strongly correlated with PNP length (Fig. 6I). Hence, *Cfl1* haploinsufficiency causes apical actomyosin accumulation and defective spinal neurulation in a proportion of embryos.

### Actin accumulation affects adherens junctions in a RhoA-dependent manner

Actomyosin disassembly defects could affect neural tube closure through disturbance of the stereotypical pattern of neural plate bending, in which the MHP and paired DLHPs are necessary for spinal closure (Greene et al., 1998; Shum and Copp, 1996; Ybot-Gonzalez et al., 2002). Inhibitor-treated and *Cfl1* mutant embryos with delayed PNP closure delay had relatively normal DHLPs (Fig. 2A, Fig. 3C, Fig. 4B and Fig. 5C; supplementary material Tables S2, S3). In contrast, we noted a reproducible lack of MHP bending in *Cfl1^−/−^* embryos and in those treated with Y27632 or Jasp, specifically at the 20–24 somite stage but not at the 15–19 somite stage (supplementary material Tables S2, S3). Hence, a lack of focal midline neuroepithelial bending could mediate the effects of actomyosin accumulation at later stages of spinal neurulation.

**Fig. 7.**
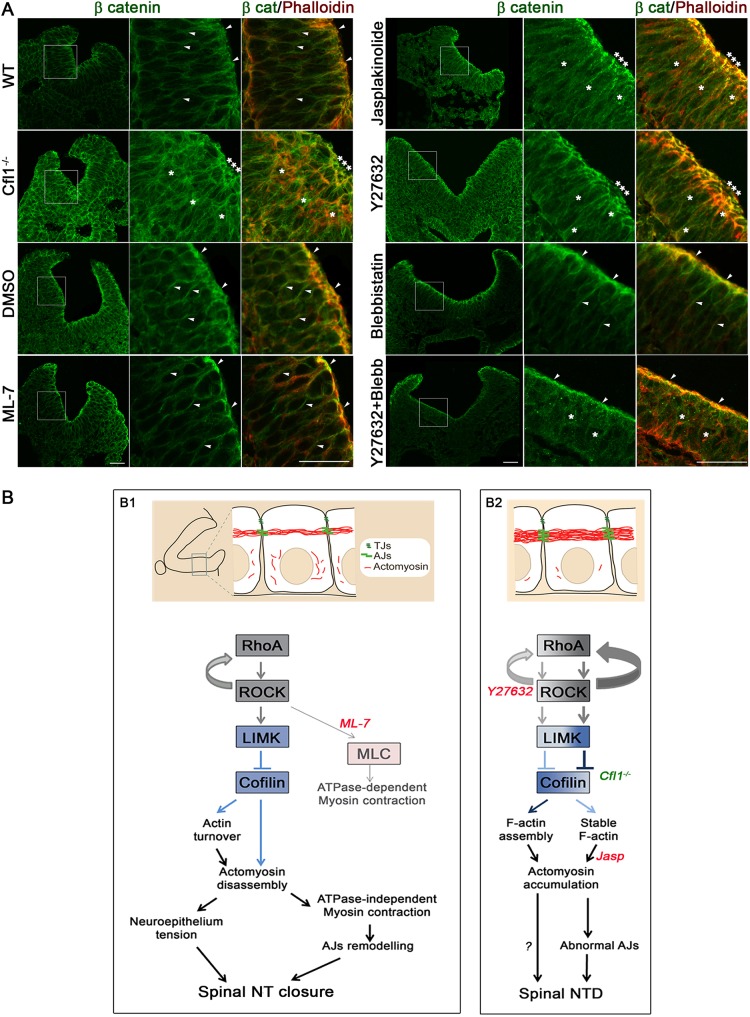
**Contribution of adherens junction disruption and summary of cytoskeletal signalling requirements for spinal neural tube closure.** (A) Immunohistochemistry (phalloidin, red; anti-β-catenin, green) reveals precise colocalization of β-catenin and phalloidin at neuroepithelial adherens junctions of E9.5 WT embryos, which is severely disrupted in *Cfl1^−/−^* littermates. Culture in Y27632 or Jasp disrupts colocalization of β-catenin and phalloidin at adherens junctions of the 15–19 somite embryos compared with DMSO controls, whereas ML-7 or Blebbistatin have no adverse effects. Culture in Y27632+Blebbistatin restores a normal staining pattern. Arrowheads, normal staining; asterisks, disrupted staining pattern. Enlargements of the boxed regions are shown for β-catenin alone and the β-catenin and phalloidin merged views. Embryos have 18–21 somites. Scale bars: 30 µm. (B) Summary of cytoskeletal signalling requirements for spinal neural tube closure, based on findings in the study. Normal closure (B1) requires signalling through RhoA, ROCK, LIMK and cofilin to regulate actin turnover and actomyosin disassembly. We suggest this is a prerequisite for both actomyosin-based neuroepithelial tension and ATPase-independent myosin contraction, the latter regulating remodelling of adherens junctions (AJs) without affecting tight junctions (TJs). Myosin ATPase-dependent contractile activity regulated by MLC phosphorylation is not limiting for spinal NT closure, as closure is not inhibited by ML-7. Failure of closure (B2) results when RhoA and ROCK signalling is inhibited by Y27632 (left arrows in B2), with apical actomyosin accumulation, probably because of enhanced cofilin activity, leading to F-actin-assembly through increased actin nucleation. Closure also fails when F-actin is stabilized (right arrows in B2), in Jasp-treated embryos or following the decrease of actin-severing activity in *Cfl1* mutants, similarly leading to actomyosin accumulation. Abnormal adherens junctions and disruption of neuroepithelial tension are suggested to result from actomyosin accumulation, and prevent spinal closure.

Another possibility is that abnormal actomyosin accumulation might affect the structure and/or function of apical junctional complexes, which are implicated in neuroepithelial morphogenesis (Nishimura and Takeichi, 2009). Indeed, cofilin plays a defined role in junctional complex remodelling (Chu et al., 2012). In wild-type embryos, β-catenin specifically localized to the lateral membranes of neuroepithelial cells with apical enrichment at adherens junctions, where it colocalized with phalloidin (Fig. 7A, WT). In contrast, *Cfl1^−/−^* embryos, and heterozygotes with large PNPs, exhibited a markedly disorganized β-catenin staining, both apically and deeper within the epithelium, which colocalized with ectopic F-actin (Fig. 7A; supplementary material Fig. S4D). Exposure of *Cfl1^−/−^* embryos to Blebbistatin in culture largely normalized β-catenin immunostaining (supplementary material Fig. S4G).

ZO1 localizes to tight junctions and showed precise immunolocalization on the apical surface of wild-type neuroepithelium (supplementary material Fig. S4E). In contrast, ZO-1 (also known as TJP1) immunostaining was more intense apically in *Cfl1^−/−^* embryos, with ectopic neuroepithelial patches that colocalized with phalloidin staining (supplementary material Fig. S4E). This ZO-1 staining defect was not normalized in *Cfl1^−/−^* embryos treated in culture with Blebbistatin (supplementary material Fig. S4H).

Immunostaining for β-catenin in Jasp- and Y27632-treated embryos revealed abnormally intense apical signal that was organized into clusters colocalizing with F-actin (Fig. 7A). In contrast, ML-7 or Blebbistatin exposure did not alter β-catenin immunolocalization apically, although staining was reduced on lateral membranes. Embryos cultured in Y27632 plus Blebbistatin exhibited a β-catenin distribution resembling that in DMSO controls (Fig. 7A). ZO-1 immunostaining was confined to the apical surface of the neuroepithelium in all cultured embryos, with no discernible abnormalities in inhibitor-treated embryos (supplementary material Fig. S4F). Hence, adherens junctions are dramatically disrupted in *Cfl1^−/−^* embryos and, less severely, when ROCK signalling and F-actin disassembly are inhibited. Tight junctions are defective in *Cfl1* mutants but not after inhibitor treatment. This suggests that actin turnover is essential in enabling adherens junctions remodelling as the spinal neuroepithelium undergoes morphogenesis.

## DISCUSSION

We have examined the cytoskeletal requirements for mouse spinal neural tube closure, using inhibitor-treated and *Cfl1* mutant embryos. The RhoA, ROCK, LIMK and cofilin signalling pathway regulates F-actin turnover in the mouse neuroepithelium and was shown to be essential for closure. Surprisingly, prevention of actomyosin cross-linking (by treatment with Blebbistatin) or F-actin assembly (by treatment with CytD or LatB) did not halt mouse spinal neurulation. In contrast, inhibiting Rho kinase (by Y27632 or HA-1100) or blocking F-actin disassembly (by Jasp and in *Cfl1* mutants) arrested closure. Actomyosin accumulation at the apical neuroepithelial surface correlated strongly with closure defects. Importantly, when the accumulated actomyosin was disassembled by Blebbistatin, neurulation disorders were rescued, wholly or partially, in Y27632- and Jasp-treated embryos and in *Cfl1* mutants. In contrast, ML-7, which inhibits myosin II contractile activity, was unable to rescue the closure defects. These findings suggest that apical actomyosin accumulation is causally related to failure of neural tube closure.

### Requirement for precise regulation of RhoA, ROCK, LIMK and cofilin signalling

Apical actomyosin accumulation and closure failure resulted from either ROCK inhibition or *Cfl1* mutation. However, inhibition of ROCK, by diminishing the sequential phosphorylation of LIMK and cofilin (Bamburg, 1999), produces abundant active cofilin whereas *Cfl1* mutants lack cofilin activity. These findings suggest a model (Fig. 7B) in which actin turnover in the neuroepithelium, and spinal neural tube closure, depend on finely tuned signalling through the RhoA, ROCK, LIMK and cofilin pathway. Cofilins sever actin filaments at low-cofilin-binding density whereas, at high concentration, cofilin can nucleate actin assembly through monomer binding (Andrianantoandro and Pollard, 2006; Ghosh et al., 2004). Cofilin depletion leads to actomyosin accumulation through a mechanism in which cofilin competitively inhibits myosin II binding to F-actin (Wiggan et al., 2012). Hence, increased active cofilin in Y27632-treated embryos and its absence in *Cfl1* mutants can both generate stable F-actin in the neuroepithelium. We conclude that mouse spinal neurulation requires precisely regulated ROCK signalling to maintain a balance between cofilin-mediated actin severing and nucleation, ensuring actin turnover and actomyosin disassembly at the apical neuroepithelium.

### Mechanisms of faulty neurulation in embryos with apical actomyosin accumulation

The process of elevation and dorsal apposition of the spinal neural folds in mice involves the formation of focal bending sites, at the midline and dorsolaterally (Greene et al., 1998; Ybot-Gonzalez et al., 2002, 2007a). Between the sites of bending, the elevating neural folds appear strikingly straight (Shum and Copp, 1996), suggesting that the generation and maintenance of overall neuroepithelial tension is also important for normal neural fold morphology. The majority of embryos with faulty closure exhibited apparently normal DLHPs, although MHPs were consistently absent from embryos with delayed closure at the 20–24 somite stage. Hence, although faulty midline bending might contribute to the later stages of closure delay, it seems unlikely that accumulation of actomyosin initiates closure delay through interference with focal bending. More likely, disturbance of neuroepithelial tension might be the primary mechanism by which Y27632- and Jasp-treatment, and *Cfl1* mutation, leads to delay in PNP closure.

Is reduced or enhanced neuroepithelial tension likely to be more disruptive of neurulation? CytD- and Blebbistatin-treated embryos exhibit ‘floppy’ neural folds (Ybot-Gonzalez and Copp, 1999) (supplementary material Table S2), and yet still progress in spinal neurulation. This suggests that diminished neuroepithelial tension is compatible with closure, provided the MHP and DLHPs are formed. Interestingly, embryos exposed to ML-7 did not exhibit floppy neural folds (supplementary material Table S2), suggesting that ATPase-dependent myosin II contractility is unlikely to be limiting for generation of neuroepithelial tension. Alternatively, ATPase-independent myosin contraction could play a role. In other systems, actomyosin ring contraction can result from dynamic de- and re-polymerization of actin filaments, independently of ATPase activity (Henson et al., 2002; Pelham and Chang, 2002; Sun et al., 2010). Hence, regulated assembly and disassembly of apical F-actin, mediated by ROCK and cofilin signalling, could be an important mechanism for generating neuroepithelial tension during spinal neurulation. We propose, therefore, that accumulation of actomyosin might generate excessive neuroepithelial tension, rendering the neural plate stiff and resistant to morphogenesis, despite the presence of MHP and DLHPs.

### Adherens junctions and spinal neurulation

A further aspect of neuroepithelial tension maintenance likely involves adherens junctions, which were consistently disturbed in neuroepithelia with actomyosin accumulation. Apical junctional complexes regulate epithelial integrity, polarity and dynamic intercellular movements during morphogenesis (Nishimura and Takeichi, 2009). In chick neurulation, PCP- and ROCK-mediated neuroepithelial cellular rearrangement depends on myosin cables that connect junctional complexes of adjacent cells (Nishimura et al., 2012), whereas adherens junctions remodelling is also strongly dependent on RhoA and ROCK activity in other systems (Fang et al., 2008; Sahai and Marshall, 2002). In *Drosophila* amnioserosa cells, actin stabilization with Jasp inhibited the redistribution of adherens junctions and increased the number of adherens junctions (Goldenberg and Harris, 2013). It seems likely, therefore, that the abnormal adherens junctions we observed in embryos with defective spinal neurulation resulted from disruption of the dynamic actomyosin assembly–disassembly cycle, and contributed to faulty neuroepithelial tension in embryos with faulty PNP closure.

### Myosin dynamics and neural tube closure

Blebbistatin blocks myosin II in an actin-detached state (Kovacs et al., 2004), thereby inhibiting two of its key functions, ATP-binding required for motor activity and actin cross-linking required for development of tension, independently of MLC phosphorylation (Bresnick, 1999; Wang et al., 2011). Given that spinal neurulation continued in the presence of Blebbistatin, we conclude that neither of these functions is limiting for closure. In fact, enhanced actin crosslinking by myosin II could also contribute to the postulated increase in neuroepithelial tension in embryos that fail in spinal closure. Of the three myosin II heavy chains, MHCB is the predominant isoform expressed in neuroepithelial cells (Wang et al., 2011) and fetal brain (Golomb et al., 2004), whereas MHCA and MHCC (also known as MYH9 and MYH14, respectively) are present at only low levels. MHCB is characterized by a high ‘duty ratio’, with strong ADP-binding and propensity to exist in a rigor state, tightly bound to actin (Rosenfeld et al., 2003; Wang et al., 2003). The striking translocation of MHCB to the apical neuroepithelial surface in embryos with ROCK inhibition (and hence increased cofilin activity) suggests that apical actin may be stabilized by MHCB, further diminishing actomyosin disassembly. Interestingly, the opposite effect, in which myosin and F-actin were found to accumulate basally in the neuroepithelium, was recently described in the cranial region of *Cfl1* mutants (Grego-Bessa et al., 2015).

How Blebbistatin acts to diminish actomyosin accumulation in the apical neuroepithelium, and rescue spinal neural tube closure, is unclear. As noted above, Blebbistatin inhibits actin cross-linking and this might help prevent actomyosin from accumulating. However, we also observed rescue when Blebbistatin was added to embryos that had been cultured for extended periods in ROCK inhibitor. This might suggest that Blebbistatin can enhance the disassembly of actomyosin, although the mechanism of this putative action is unknown.

### Stage and species variations in cytoskeletal requirement for neurulation

The onset of neural tube closure is strongly dependent on the PCP pathway in mice (Copp and Greene, 2010) and it has been shown recently that the severe PCP closure phenotype of homozygous *loop-tail* (*Vangl2*^Lp^) embryos is further exacerbated by co-inactivation of *Cfl1* or by Jasp treatment. This confirmed a role for F-actin turnover in PCP initiation at the onset of closure (Mahaffey et al., 2013). Once closure has been initiated, at the brain–spine boundary, the cranial neural plate undergoes major morphogenetic changes, starting with expansion of bi-convex neural folds, followed by dorsolateral bending, which brings the neural fold tips together in the dorsal midline (Morriss-Kay, 1981). This latter event has been found to be exquisitely sensitive to disruption of apical actin microfilaments, with cytochalasins causing exencephaly both *in vivo* and *in vitro* (Austin et al., 1982; Morriss-Kay and Tuckett, 1985; Wiley, 1980). We observed cranial neural tube defects in embryos cultured in Blebbistatin and LatB (data not shown), suggesting a general requirement for contractile actomyosin in cranial neural folding. Following completion of cranial neurulation, the spinal neural tube ‘zips up’ along the body axis to complete closure of the primary neural tube, and this process has strikingly different cytoskeletal requirements from cranial closure where regulation of neuroepithelial tension is required with a key role for ATPase-independent actomyosin dynamics.

In contrast to mammals, lower vertebrates appear to be more completely reliant on ATPase-dependent actomyosin contraction for neural tube closure. Chick embryos exposed to Blebbistatin exhibit severe neural tube defects due to defective apical constriction (Kinoshita et al., 2008) whereas myosin II knockdown in *Xenopus laevis* impairs neural plate bending (Rolo et al., 2009). These differences between animal groups might reflect the somewhat ‘simpler’ pattern of neurulation events in birds and amphibians where closure of brain and spine are relatively homogeneous in timing and morphology compared with mammals, where the events along the body axis cover a long developmental period and are morphologically extremely diverse (Morriss-Kay, 1981).

## MATERIALS AND METHODS

### Breeding and genotyping of mouse strains

Mouse procedures were performed under the UK Animals (Scientific Procedures) Act 1986 and ‘Responsibility in the Use of Animals for Medical Research’ (Medical Research Council, 1993). *Cfl1*-knockout mice were bred and genotyped as described previously (Gurniak et al., 2005). Non-mutant embryos were from random-bred CD1 mice.

### Embryo collection and culture

After overnight mating, pregnant females were killed at E8.5–E9.5 days of gestation (E0.5 is noon on the day of the copulation plug). Embryos were dissected in Dulbecco's modified Eagle's medium (Invitrogen) containing 10% fetal bovine serum (Sigma). Following whole embryo culture (Copp et al., 2000), or immediately after dissection of non-cultured embryos, somites were counted and PNP length was measured using an eyepiece graticule on a Zeiss SV11 stereomicroscope. Embryos for immunostaining were fixed for 1 h in 4% paraformaldehyde (PFA) in phosphate-buffered saline (PBS), embedded in 7.5% gelatin in 15% sucrose in PBS and cryosectioned. Embryos for protein extraction were rinsed twice in ice-cold PBS, snap frozen on liquid N_2_ and stored at −80°C. For western blotting, F-actin and G-actin ratio and Rho activation assays, PNP and ‘body’ fragments were generated by cutting at the rostral border of the most recently formed somite pair. Both fragment types were assayed separately.

### Chemical inhibitors

Inhibitors were prepared as stock solutions in DMSO and stored frozen as follows: Cytochalasin D (C8273, Sigma, 100 mg/ml), Latrunculin B (catalogue no. 428020, Calbiochem, 25 mg/ml), Blebbistatin (catalogue no. 203390, Calbiochem, 75 mg/ml), ML-7 (catalogue no. 12764, Sigma, 10 mM), Y27632 (catalogue no. 688000, Calbiochem, 5 mM), HA-1100 (catalogue no. 390602, Calbiochem, 10 mM), Jasplakinolide (catalogue no. 420107, Calbiochem, 1 mM). Immediately prior to use, stocks were diluted with DMSO and added to embryo culture serum to a maximum of 0.1% (v/v). Culturing was performed in the dark to avoid photo-inactivation.

### Protein extraction and western blotting

Proteins were extracted from frozen unfixed tissue by sonication in RIPA buffer (1% Nonidet P-40,150 µM NaCl, 10 µM Tris-HCl pH 8, 1× complete protease inhibitor cocktail, in PBS). Tissue lysates were cleared of unbroken cells by centrifugation (2000 ***g***), electrophoresed on 10% or 4–12% Bis-Tris gels (NuPage, Invitrogen) and transferred onto PVDF membrane (XCell II Blot Module, Invitrogen). Primary antibodies for immunodetection are described in supplementary material Table S4. Detection was by horseradish-peroxidase-conjugated secondary antibodies (DAKO), followed by development with the ECL prime western blotting detection system (GE Healthcare). Autoradiographs were scanned using a GS-800 Imaging Densitometer (Bio-Rad) and analysed with Quantity One software (Bio-Rad).

### F-actin and G-actin ratio and Rho activation assays

The ratio of free monomeric actin (G-actin) to ﬁlamentous actin (F-actin) was determined by fractionation. Cells were lysed in ice-cold PHEM buffer (60 mM Pipes, 20 mM HEPES, 10 mM EGTA, 2 mM MgCl_2_, pH 7.0, 1% Triton X-100). Cleared lysates (2000 ***g***) were centrifuged at 10,000 ***g*** to separate Triton-X-100-soluble G-actin from Triton-X-100-insoluble F-actin. Supernatant containing G-actin was collected, and the F-actin pellet was washed twice in cold PHEM buffer and suspended in SDS buffer. Fractions were proportionally loaded onto SDS-polyacrylamide gels, electrophoresed and transferred onto PVDF membrane for probing with an anti-actin antibody (A5316, Sigma). Densitometric quantification of the western blot was used to determine the G-actin versus F-actin content.

Activated RhoA was determined by using the RhoA G-LISA Activation Assay kit (catalogue no. BK124, Cytoskeleton Inc.) according to the manufacturer's instructions. Active GTP-bound RhoA in protein lysates was bound to RhoA-GTP-binding proteins linked to the wells of a 96-well plate, whereas inactive GDP-bound forms were removed during wash steps. GTP-bound Rho was detected by indirect immunofluorescence using anti-RhoA antibody. Absorbance was measured at 490 nm using a microplate spectrophotometer reader (BioTek synergy HT). Total RhoA was determined by an ELISA assay (catalogue no. BK150, Cytoskeleton Inc.). Assays were performed with duplicate measures.

### Immunohistochemistry

Gelatin was removed from cryosections by incubation in PBS for 30 min at 37°C. Samples were blocked and permeabilized in 10% sheep serum and 0.1% Tween-20 in PBS, incubated with primary antibody overnight at 4°C, rinsed in PBS, and incubated with secondary antibody for 1 h at room temperature. For F-actin, sections were incubated in phalloidin (Alexa-Fluor-568–phalloidin, A12380, Life Technologies) for 1 h at room temperature. Samples were washed with 4′,6-diamidino-2-phenylindole (DAPI) and mounted in Mowiol 4-88 mounting medium (Sigma-Aldrich, MO; prepared with glycerol and 0.2 M Tris-HCl pH 6.8). Primary antibodies are described in supplementary material Table S4. The secondary antibody was Alexa-Fluor-488-conjugated goat anti-rabbit-IgG (A11070, Life Technologies).

### Cell proliferation and cell death analysis

Immunostaining was performed for phosphorylated histone H3 (Millipore) and cleaved caspase 3 (Cell Signaling) on 12-μm transverse crysections at the axial level of the closing spinal neural tube (17–19 somites). Fluorescence images were collected using a Zeiss Axiophot microscope with Leica DC500 camera and FireCam software. Images were analysed using the Cell Counter plugin of the ImageJ software. Phosphorylated-histone-H3- or caspase-3-positive cells were counted and expressed as a percentage of the total cell number visualized by DAPI staining. Three embryos per treatment were analysed and, for each embryo, cells from at least three sections were counted.

### MHP and DLHP scoring

During spinal neural plate bending, the MHP is defined as an acute (focal) midline bend in the neural plate, present during the early stages (Modes 1 and 2) of mouse spinal neurulation. At the latest stage (Mode 3), an MHP is absent: the midline neural plate is not focally bent, but gradually or uniformly curved, or even flat (Shum and Copp, 1996). These criteria were used to score the MHP as either present or absent, after inspection of serial sections throughout the PNP. For examples of MHP-positive neural plates, see Fig. 2A (DMSO) and Fig. 3C (DMSO), whereas MHP-negative neural plates are seen in Fig. 2A (Blebb) and Fig. 3C (Jasp). DLHPs like the MHP are characterized by an acute focal bend of the neural plate, present during late stages of spinal neurulation (Modes 2 and 3) but not at the earliest stage (Mode 1) (Shum and Copp, 1996). In transverse section, DLHPs are always centred at the point where the neural plate transitions from contact with surface ectoderm, dorsolaterally, to contact with paraxial mesoderm, ventromedially. These criteria were used to score DLHPs as either present or absent, after inspection of serial sections throughout the PNP. For examples of DHLPs, see Fig. 3C, Fig. 5C and Fig. 6C.

### Confocal microscopy

Labelled cells in immunohistochemistry or phalloidin-stained sections were examined and quantified by epifluorescence on an inverted LSM710 confocal system mounted on an Axio Observer Z1 microscope (Carl Zeiss Ltd, UK). Images were acquired at room temperature using a 63× oil immersion objective. The thickness of optical sections was set at 0.2 to 0.9 μm. The Alexa Fluor 488 dye was excited by a 488 nm line of an Argon laser and Alexa Fluor 568 by a 561 nm diode laser. *Z-*projections of confocal stacks were created in ImageJ. Images were further processed in Photoshop CS3 (Adobe).

### Image analysis and quantification

Between three and five adjacent immunohistochemistry or phalloidin-stained sections were analysed per embryo. For each section and each side of the neural plate, a region of interest (ROI) was outlined, comprising the full thickness of the neural plate along ∼30% of the dorsoventral extent of one hemi-plate. The fluorescence intensity within the ROI was measured using the ImageJ function ‘Plot Profile’ integrated in custom written ImageJ macro number 1. In this macro, ImageJ plot profile function displays a ‘column average plot’, where the *x*-axis represents the basal-apical distance through the ROI, and the *y*-axis represents the vertically averaged pixel intensity of the ROI. Each curve was normalized to a range of 0–100 along the basalapical axis (*x*-axis). Once normalized, the intensity values (*y*-axis) were interpolated (integers between 0 and 100 for the X axis) using a custom-written Matlab script (macro number 2) to allow statistical analysis and comparison between samples. For some phalloidin-stained sections, the intensity values (*y*-axis) were also normalized using macro number 1. Macros are available upon request. See also supplementary material Fig. S1E.

### Statistical analysis

Statistical tests were performed using SigmaStat v3.5 (Systat Software Inc). At least three independent experiments were performed for each assay.

## Supplementary Material

Supplementary Material
