# Supplementary material for: Rho-kinase-dependent actin turnover and actomyosin disassembly are necessary for mouse spinal neural tube closure
Source: J Cell Sci. 2015 Jul 15;128(14):2468–81. doi: 10.1242/jcs.164574 (PMC4510849; doi:10.1242/jcs.164574)
Supplement: Supplementary Material [file supp_128_14_2468__index.html]

Supplementary Material 

# Rho kinase-dependent actin turnover and actomyosin disassembly are necessary for mouse spinal neural tube closure

## JCS164574 Supplementary Material

- Supplementary Material
