# Supplementary material for: Rho-kinase-dependent actin turnover and actomyosin disassembly are necessary for mouse spinal neural tube closure
Source: J Cell Sci. 2015 Jul 15;128(14):2468–81. doi: 10.1242/jcs.164574 (PMC4510849; doi:10.1242/jcs.164574)
Supplement: Supplementary Material [file supp_jcs.164574_JCS164574supp.pdf]

Supplementary figure legends

Figure S1. Effect of cytoskeletal inhibitors on neural tube closure, and method of intensity profile analysis

(**A-B**) Mean PNP length (mm ± SEM) after treatment with different inhibitors for 5-6 h (A) or 18-20 h (B). P-values are from pairwise t-tests compared with DMSO controls, following 1-way ANOVA ($p < 0.05$).

(**C**) Embryos exposed to 12.5, 20 or 50 μM Blebbistatin for 5-6 h undergo PNP closure at the same rate as DMSO controls (best fit linear regression lines plotted).

(**D**) Graph (top) and table (below) showing PNP length at 20-24 somites following 16-17 h culture is not significantly different from DMSO control after exposure to 60 μM Blebbistatin (a higher concentration than the 50 μM used elsewhere in the study). Similarly, a combination of 50 μM Blebbistatin + 0.05 μg/mL CytD, or 50 μM Blebbistatin + 50 μM ML-7, does not yield PNP lengths significantly different from DMSO controls (P-values in table = 0.694 for 1-way ANOVA; $p > 0.05$).

(E) Method of analysing fluorescence intensity of Phalloidin and anti-MHCB staining on sections through the neural plate. (E1) Fluorescence intensity was measured along the basal-to-apical (X) axis of each region of interest. Image J function "Plot Profile" and custom written ImageJ macro #1 were used to provide a 'column average profile' for the region of interest. (E2) Average profile curves were normalised to a range of 0-100 along the basal-to-apical axis. (E3) Intensity values were interpolated (integers between 0 and 100 for the X axis) using a custom written Matlab script (macro #2), enabling statistical analysis and comparison between samples.

Figure S2. Effect of cytoskeletal inhibitors on Phalloidin intensity, F/G actin fractions and neural tube closure

(A) Intensity profile scans of Phalloidin staining along the basal-to-apical axis of the neuroepithelium. Basal surface = 0; apical surface = 100 (arbitrary units, a.u.). The maximum intensity is normalised to 100%. Note the reproducibility of the basally extended Phalloidin staining in individual Y27632-treated embryos compared with DMSO controls.

(B) Biochemical fractionation shows increased F-actin and reduced G-actin in Y27632-treated embryos, but dramatically reduced F-actin and increased G-actin in CytD-treated embryos (** $p < 0.001$ compared with DMSO control).

(C, D) Intensity profile scans of Phalloidin staining (C) and MHCB (D) along the basal-to-apical axis of the neuroepithelium. Note the very low intensity of Phalloidin and MHCB in embryos treated with Blebbistatin + CytD or Blebbistatin + ML-7, consistent with severe reduction of neuroepithelial actomyosin by these inhibitors.

(**E**) PNP length (mm ± SEM) is significantly increased after treatment with Jasp for 18-20 h to the 15-19 or 20-24 somite stage. P-values are from t-tests compared with DMSO controls.

(**F**) Biochemical fractionation shows increased F-actin and reduced G-actin in Jasp-treated embryos compared with DMSO control embryos (** $p < 0.001$).

(**G**) Intensity profile scans of Phalloidin staining along the basal-to-apical axis of the neuroepithelium in embryos treated with Jasp or DMSO. The maximum intensity is normalised to 100%. Note the reproducibility of the basally extended Phalloidin staining in individual Jasp-treated embryos, compared with DMSO controls.

(**H, I**) Quantitation of cell proliferation by % phospho-histone H3 positive nuclei (H) and programmed cell death by % cleaved caspase 3 positive cells (I) in embryos cultured in Y27632 or Jasp for 18-20 h. There is no statistical difference between either treatment group and DMSO control embryos.

Figure S3. Effects of cytoskeletal inhibitors on neural tube closure and F/G actin fractions, and rescue of Y27632-induced and Jasp-induced defects by Blebbistatin

(**A**) Mean PNP length (mm ± SEM) after treatment with 50 μM ML-7 or DMSO for 5-6 h. There is no significant difference between ML-7 and DMSO-treated embryos at either 15-19 or 20-24 somites. P-values are from t-tests compared with DMSO controls.

(**B**) Embryos exposed to varying concentrations of ML-7 for 5-6 h undergo PNP closure at a similar rate as DMSO-treated controls (best fit linear regression lines plotted).

(**C**) Mean PNP length (mm ± SEM) after treatment with DMSO, Y27632, Y27632 + Blebbistatin, Y27632 + ML-7 for 15-18h. Note the rescue of PNP closure delay in Y27632-treated embryos by Blebbistatin, but not by ML-7. P-values are from pairwise t-tests compared with DMSO controls, following 1-way ANOVA ($p < 0.05$)

(**D, E**) Mean PNP length (mm ± SEM) after Y27632 treatment for 5-6 h followed by Blebbistatin for 13-15 h (D) or treatment with Y27632 + Blebbistatin for 5-6 h to the 15-19 or 20-24 somite stage (E). In both cases, PNP length does not differ from DMSO controls. P-values are from t-tests compared with DMSO controls.

(**F, H**) Mean PNP length (mm ± SEM) after culture for 5-6 h (Short Term Culture, STC) or 15-18 h (Long Term Culture, LTC), shown graphically (F) and in table form (H, lower). Y27632 produces enlarged PNPs (pink symbols) but this effect is reversed if embryos are cultured in rat serum alone after removal ('washout') of Y27632 (STC Y27632 then RS). However washout with rat serum after longer exposure to Y27632 does not restore PNP length (LTC Y27632 then RS) whereas subsequent culture in Blebbistatin rescues closure (LTC Y27632 then Blebb). P-values are from t-tests compared with DMSO controls.

(**G, H**) Mean PNP length (mm ± SEM) after culture in Y27632 followed by Blebbistatin, shown graphically (G) and in table form (H, upper). The effect of Y27632 in producing enlarged PNPs (pink symbols) is reversed when Blebbistatin is added to either short-term (STC Y27632 then Blebb) or long-term cultures (LTC Y27632 then Blebb). The same result is observed in embryos reaching three different somite stages.

(**I**) Biochemical fractionation shows similar a F/G ratio in embryos treated with a mixture of Y27632 and Blebbistatin after STC or LTC in Y27632 (as in G). There are no statistical differences in F/G ratio from DMSO control embryos.

(**J, K**) Exposure to a combination of 10 nM Jasplakinolide + 50 μM Blebbistatin for 15-18h does not significantly affect PNP closure compared with DMSO controls ($p > 0.05$). Compare with the marked PNP closure-delaying effect of Jasp alone in Fig. 3A and Fig. S2E.

Figure S4. Actomyosin accumulation, apical junction formation, and PNP closure in *Cofilin* mutant and inhibitor-treated embryos

(A) Mean PNP length (mm ± SEM) for wild-type (WT), *Cofilin 1*$^{+/-}$ and *Cofilin 1*$^{-/-}$ littermate embryos. Note the enlarged PNPs in *Cofilin 1* mutant embryos at 20-24 somites. P-values are from pairwise t-tests compared with WT following 1-way ANOVA ($p < 0.05$).

(**B**) Immunohistochemistry (Phalloidin, red; anti-MHCB, green) in WT and *Cofilin 1*$^{-/-}$ mutants at 16 somites, preceding onset of PNP closure delay. Note apical accumulation of actomyosin (asterisks) in the mutant neuroepithelium. Scale bar: 30 μm in all panels

(**C, D**) Apical accumulation of actomyosin and disruption of adherens junctions (Phalloidin, red; anti-MHCB, green in B; β-catenin, green in C) in *Cofilin 1*$^{+/-}$ embryos with a large PNP (lower panels) compared with those with a small PNP (upper panels). The latter exhibit relatively normal actomyosin and β-catenin immunostaining.

(**E**) Immunohistochemistry (Phalloidin: red; anti-ZO1: green) in the neuroepithelium of E9.5 WT (upper panel) and *Cofilin 1*$^{-/-}$ (lower panel) embryos. ZO1 immunostaining is disrupted in

Cofilin 1 mutants compared with WT. Arrowheads: normal staining; asterisks: disrupted staining pattern. Right panels show enlargements of boxed regions. Scale bar: 30 µm in all panels.

(**F**) Immunohistochemistry (anti-ZO1: green) in the neuroepithelium of E9.5 embryos following culture with inhibitors for 6 or 18 h. ZO1 immunostaining is unaffected in inhibitor-treated embryos compared with DMSO controls. Embryos have 18-21 somites. Arrowheads: normal staining. Scale bar: 30 µm in all panels.

(**G**) Immunohistochemistry (Phalloidin, red; anti-MHCB, green, middle; anti-β-catenin, green, right) reveals a less severely disrupted actomyosin distribution in *Cofilin 1* mutant embryos exposed to Blebbistatin (G) compared with untreated *Cofilin 1* mutants (compare with Figs 6C, S4B). Blebbistatin treatment is also able to restore normal β-catenin staining (compare with Fig. 7A).

(**H**) In contrast, *Cofilin 1* embryos exposed to Blebbistatin show abnormal ZO1 staining, indicating that TJ structure is not rescued (compare with Fig. S4E). Asterisks: abnormal immunostaining.

A

	15-19 somites			20-24 somites		
	mean	n	P value	mean	n	P value
DMSO	0.37 ± 0.05	7		0.17 ± 0.04	8	
CytD	0.42 ± 0.05	6	P = 0.45	0.28 ± 0.04	10	P = 0.53
Blebb	0.33 ± 0.03	13	P = 0.364	0.23 ± 0.04	9	P = 0.45
Y27632	0.68 ± 0.03	14	P < 0.001	0.46 ± 0.04	9	P < 0.001

B

	15-19 somites			20-24 somites		
	mean	n	P value	mean	n	P value
DMSO	0.29 ± 0.03	8		0.20 ± 0.02	15	
Blebb	0.33 ± 0.03	3	P = 0.504	0.17 ± 0.02	7	P = 0.369
CytD	0.28 ± 0.01	4	P = 0.858	0.17 ± 0.04	3	P = 0.536
LatB	0.35 ± 0.09	5	P = 0.510	0.08 ± 0.04	7	P = 0.013
Y27632	0.57 ± 0.05	21	P < 0.001	0.44 ± 0.06	12	P < 0.001
HA-1100	0.57 ± 0.04	3	P < 0.001	0.48± 0.15	3	P < 0.05

C

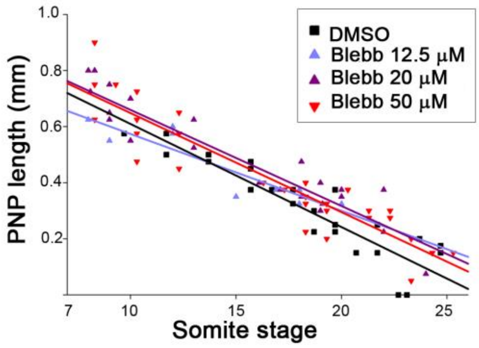

D

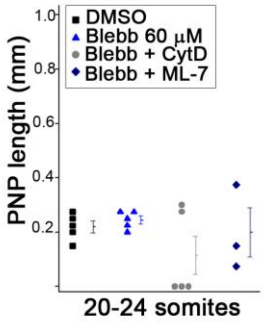

	mean	n	P value
DMSO	0.22 ± 0.02	5	
Blebb 60 uM	0.24 ± 0.01	5	P = 0.694
Blebb+CytD	0.11 ± 0.07	5	P= 0.694
Blebb+ML-7	0.2 ± 0.09	3	P= 0.694

E

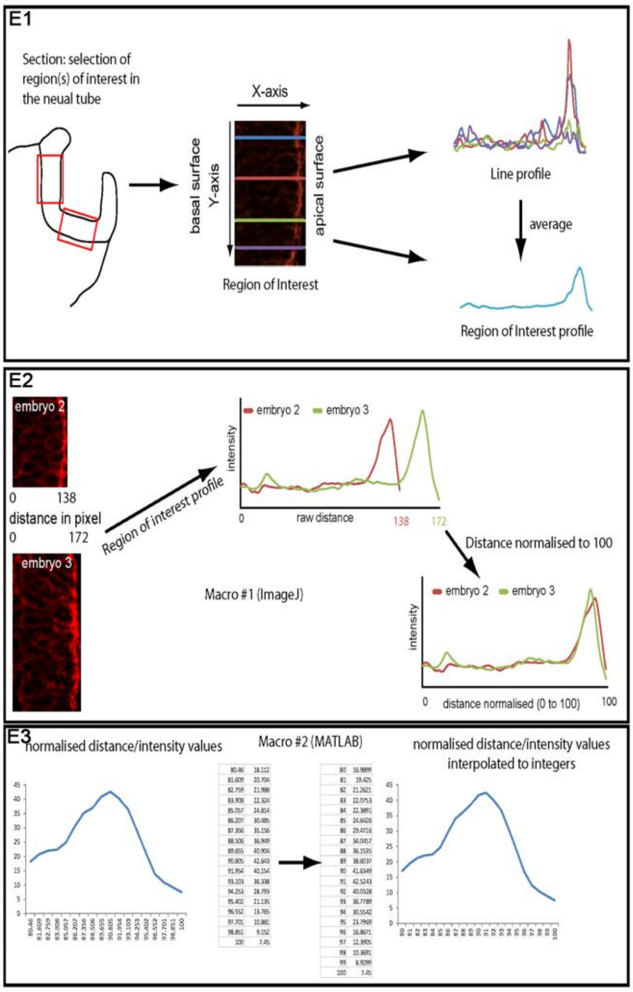

Figure S1

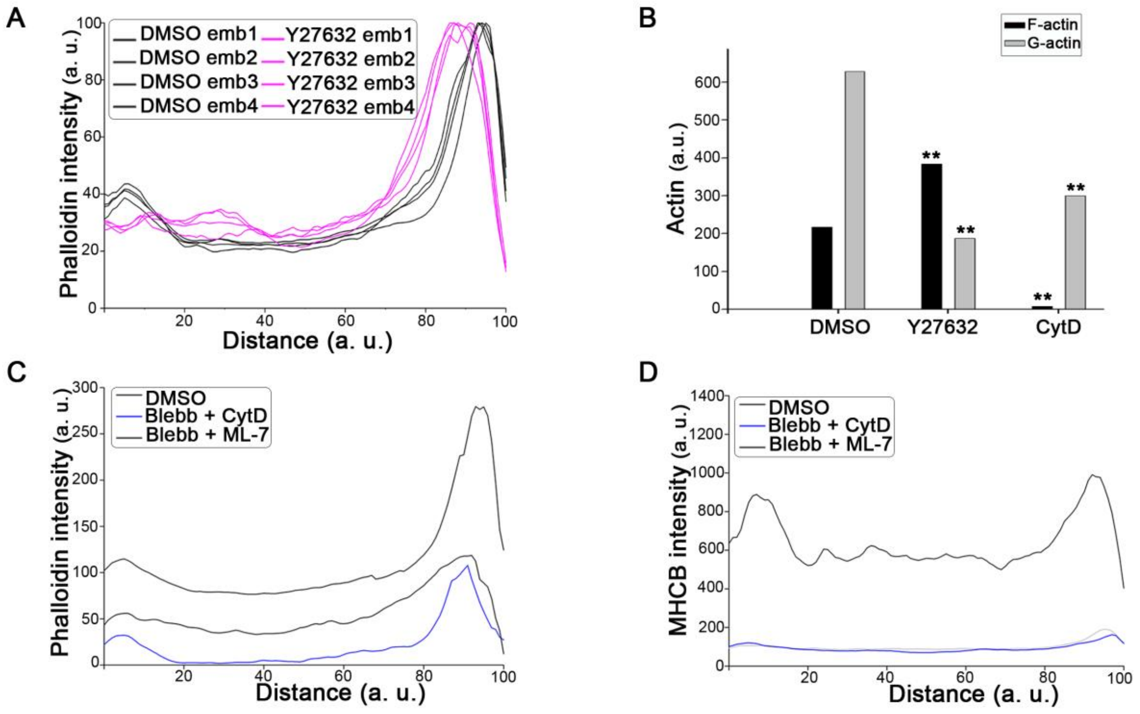

E

	15-19 somites			20-24 somites		
	mean	n	P value	mean	n	P value
DMSO	0.28 ± 0.06	11		0.22 ± 0.05	17	
Jasplakinolide	0.50 ± 0.03	16	P < 0.05	0.40 ± 0.03	16	P < 0.05

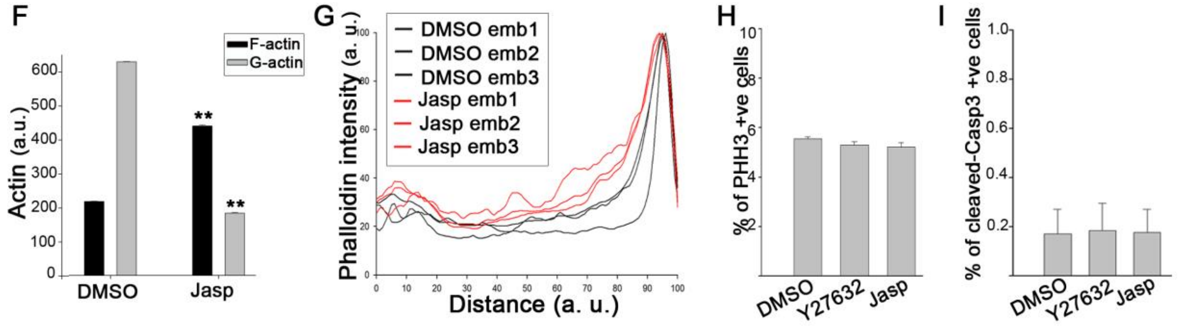

Figure S2

A

	15-19 somites			20-24 somites		
	mean	n	P value	mean	n	P value
DMSO	0.42 ± 0.05	6		0.17 ± 0.04	10	
ML7	0.46 ± 0.05	6	P = 0.623	0.29 ± 0.04	12	P = 0.059

B

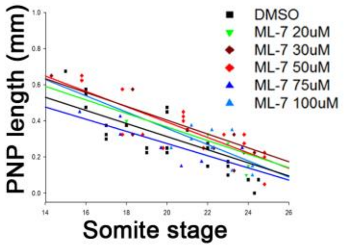

C

	mean	n	P value
DMSO	0.19 ± 0.03	13	
Y27632	0.44 ± 0.04	10	P < 0.05
Y27632+Blebb	0.25 ± 0.02	10	P = 0.234
Y27632+ML-7	0.46 ± 0.03	14	P < 0.05

D

	mean	n	P value
DMSO	0.29 ± 0.07	6	
Y27632 then Blebb	0.23 ± 0.06	5	P = 0.582

E

	15-19 somites			20-24 somites		
	mean	n	P value	mean	n	P value
DMSO	0.41 ± 0.03	7		0.17 ± 0.05	8	
Y27632+Blebb	0.40 ± 0.04	7	P = 0.801	0.27 ± 0.04	9	P = 0.102

F

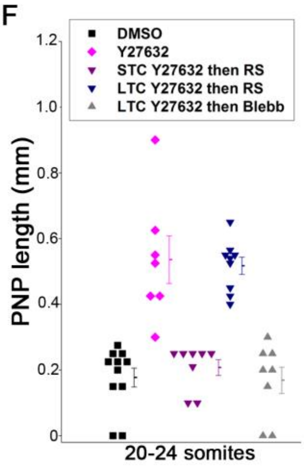

G

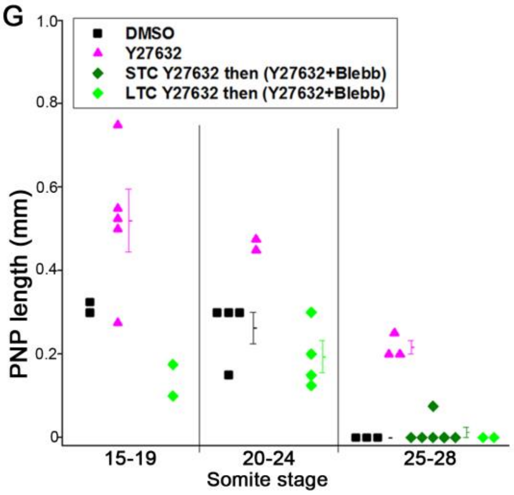

I

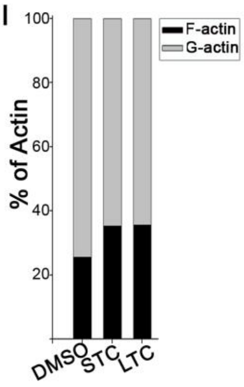

H

	15-19		20-24		25-28	
	mean	n	mean	n	mean	n
DMSO	0.31	2	0.26 ± 0.04	4	0.00 ± 0.00	3
Y27632	0.52 ± 0.08	5	0.46	2	0.22 ± 0.02	3
STC Y27632 then (Y27632+Blebb)					0.01 ± 0.01	5
LTC Y27632 then (Y27632+Blebb)	0.14	2	0.19 ± 0.04	4	0	2

	mean	n	P value
DMSO	0.18 ± 0.03	11	
Y27632	0.54 ± 0.07	7	P < 0.05
STC Y27632 then RS	0.21 ± 0.02	8	P = 0.420
LTC Y27632 then RS	0.52 ± 0.03	9	P < 0.05
LTC Y27632 then Blebb	0.17 ± 0.04	8	p = 0.967

J

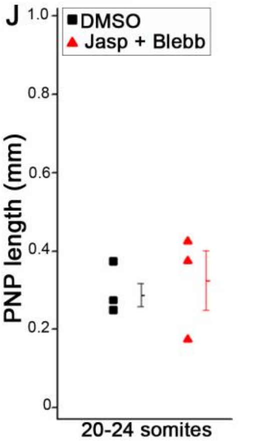

K

	mean	n	P value
DMSO	0.29 ± 0.03	3	
Jasp+Blebb	0.32 ± 0.07	3	P = 0.784

Figure S3

A

	15-19 somites			20-24 somites		
	mean	n	P value	mean	n	P value
Cofilin $1^{+/+}$	0.59 ± 0.03	9		0.34 ± 0.02	14	
Cofilin $1^{+/-}$	0.59± 0.06	3	P = 0.960	0.40 ± 0.03	10	P = 0.178
Cofilin $1^{-/-}$	0.56 ± 0.04	6	P = 0.560	0.66 ± 0.05	5	P < 0.001

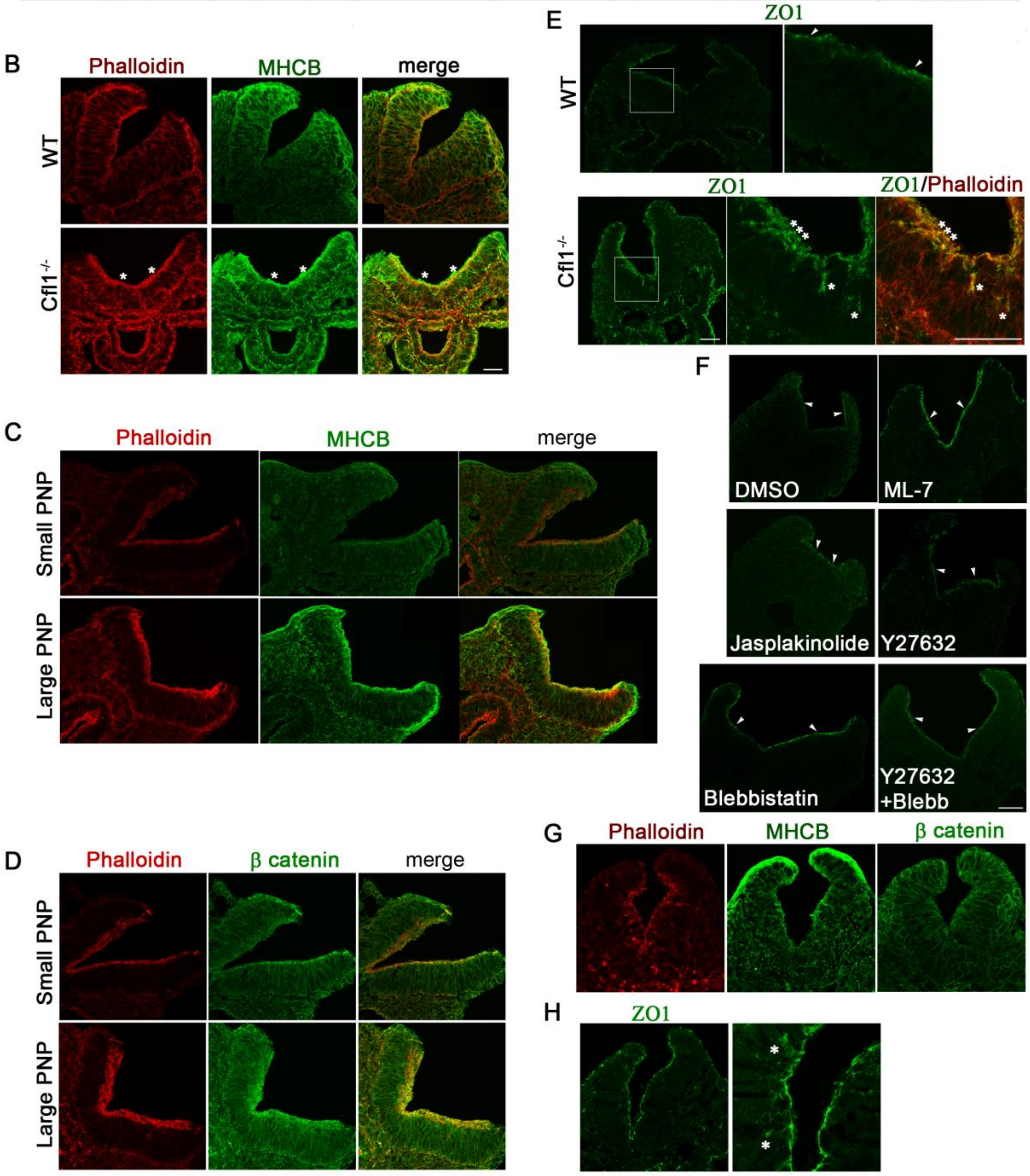

Figure S4

Table S1.

Dose-response experiments used to select inhibitor concentrations for embryo culture [a]

Inhibitor	Action	Concentration [b]	Yolk sac circulation	Heart beat	Normal overall morphology [c]
**CytD**	Blocks actin polymerisation	**0.05 µg/mL**	22/23	23/23	23/23
		0.1 µg/mL	1/6	3/6	1/6
**LatB**	Blocks actin polymerisation	500 nM	3/3	3/3	3/3
		1 µM	4/4	4/4	4/4
		2.5 µM	3/3	3/3	3/3
		5 µM	3/3	3/3	3/3
		**10 µM**	11/13	11/13	11/13
		20 µM	1/4	2/4	2/4
**Blebbistatin**	Inhibits myosin ATPase activity	12.5 µM [d]	8/9	9/9	9/9
		20 µM [d]	24/26	26/26	24/6
		**50 µM** [d]	28/32	32/32	29/32
		75 µM [d]	1/3	2/3	1/3
		100 µM [d]	0/4	0/4	0/4
**Y27632**	Inhibits Rho kinase ROCK	**5 µM** [e]	42/43	43/43	43/43
		10 µM [e]	2/6	4/6	2/6
**HA-1100**	Inhibits Rho kinase ROCK	10 µM	4/4	4/4	4/4
		**20 µM**	9/9	9/9	9/9
		30 µM	0/3	0/3	0/3
		50 µM	0/3	0/3	0/3
**ML-7**	Inhibits myosin light chain kinase MLCK	5 µM	3/3	3/3	3/3
		10 µM	3/3	3/3	3/3
		20 µM	5/6	6/6	6/6
		30 µM	10/10	10/10	10/10
		**50 µM**	17/18	18/18	18/18
		75 µM	7/7	7/7	7/7
		100 µM	8/9	9/9	8/9
		125 µM	1/5	2/5	1/5
**Jasplakinolide**	Blocks F-actin depolymerisation	**10 nM** [e]	31/32	32/32	32/32
		50 nM [e]	0/2	1/2	0/2
		100 nM [e]	0/3	0/3	0/3

[a] Embryos were cultured for 18-20h to the 15-24 somite stage, after which presence of yolk sac circulation and heart beat were evaluated as measures of viability.

[b] Concentrations in red are those used in the experimental studies.

[c] Overall morphology was scored with the following defects considered 'abnormal': non-smooth and round yolk sac, non-well formed branchial arches and maxillary, abnormal shaped somites.

[d] The heart was bigger and the beating slower in Blebbistatin-treated embryos at all concentrations tested

[e] Ssome embryos treated with Y27632 or jasplakinolide did not complete axial rotation

Table S2.

Number of embryos that exhibited MHP and DLHP bending in the PNP following culture in inhibitors

	Bending regions		Non-bending regions
	MHP [a]	DLHP [a]	Rigidity [b]
**Short cultures (5-6 h)**			
**15-19 somites**			
DMSO	20/20	20/20	normal, straight
CytD	6/6	6/6	loss of rigidity/buckle
Blebbistatin	13/13	13/13	loss of rigidity/buckle
Y27632	14/14	14/14	normal, straight
Y27632 + Blebbistatin	7/7	7/7	normal, straight
ML-7	6/6	6/6	normal, straight
**20-24 somites**			
DMSO	21/26	26/26	normal, straight
CytD	10/10	10/10	loss of rigidity/buckle
Blebbistatin	8/9	9/9	loss of rigidity/buckle
Y27632	3/9	9/9	normal, straight
Y27632 + Blebbistatin	8/9	9/9	normal, straight
ML-7	11/12	12/12	normal, straight
**Long cultures (18-20 h)**			
**15-19 somites**			
DMSO	17/19	19/19	normal, straight
CytD	4/4	4/4	loss of rigidity/buckle
Blebbistatin	3/3	3/3	loss of rigidity/buckle
Y27632	20/21	21/21	normal, straight
Jasplakinolide	16/16	16/16	normal, straight
**20-24 somites**			
DMSO	39/45	45/45	normal, straight
CytD	3/3	3/3	loss of rigidity/buckle
Blebbistatin	6/7	7/7	loss of rigidity/buckle
Y27632	3/22	22/22	normal, straight
Jasplakinolide	2/16	16/16	normal, straight
Y27632 + Blebbistatin	9/10	10/10	normal, straight
Y27632 + ML-7	4/14	14/14	normal, straight

[a] Values are number with MHP or DLHP/total number of embryos analysed.

[b] Morphological assessment of non-bending regions of neural plate which are normally straight.

[c] Note that MHP bending was absent from the majority of Y327632- and Jasp-treated embryos at 20-24 somites, but not at 15-19 somites.

Table S3.

Number of *Cofilin 1* mutant embryos that exhibited MHP and DLHP bending in the PNP

	Bending regions		Non-bending regions
	MHP [a]	DLHP [a]	Rigidity [b]
**15-19 somites**			
WT	9/9	9/9	normal, straight
Cofilin 1 $^{+/-}$	3/3	3/3	normal, straight
Cofilin 1 $^{-/-}$	6/6	6/6	normal, straight
**20-24 somites**			
WT	13/14	14/14	normal, straight
Cofilin 1 $^{+/-}$	9/10	10/10	normal, straight
Cofilin 1 $^{-/-}$	2/5 [c]	5/5	normal, straight

[a] Values are number with MHP or DLHP/total number of embryos analysed.
[b] Morphological assessment of non-bending regions of neural plate which are normally straight.
[c] Note that MHP bending was absent from the majority of cofilin 1 homozygotes at 20-24 somites.

Table S4.

Primary antibodies

**Protein specificity**	**Supplier**	**Usage ***
Cofilin	sc-8441, Santa Cruz	WB
pCofilin	#3313, Cell Signaling	WB
LIMK	sc-8389, Santa Cruz	WB
pLIMK	#3841, Cell Signaling	WB
MLC	#3672, Cell Signaling	WB
pMLC	#3671, Cell Signaling	WB
GAPDH	MAB374, Millipore	WB, IHC
β-catenin	ab16051, abcam	IHC
MHCIIB	PRB-445P, Covance	IHC
ZO1	#40-2200, Life technologies	IHC
PHH3	06-570, Millipore	IHC
Cleaved-Caspase 3	#9661, Cell signaling	IHC

* WB, Western blot; IHC, immunohistochemistry
